# EGFR exon 19‐deletion aberrantly regulate ERCC1 expression that may partly impaired DNA damage repair ability in non‐small cell lung cancer

**DOI:** 10.1111/1759-7714.13253

**Published:** 2019-12-25

**Authors:** Linlin Zhang, Barun Pradhan, Lili Guo, Fanlu Meng, Diansheng Zhong

**Affiliations:** ^1^ Department of Medical Oncology Tianjin Medical University General Hospital Tianjin China; ^2^ Genome‐Scale Biology Research Program, Research Programs Unit, Faculty of Medicine University of Helsinki Helsinki Finland; ^3^ Tianjin Lung Cancer Institute Tianjin Medical University General Hospital Tianjin China

**Keywords:** DNA damage repair, EGFR exon 19 deletion, ERCC1, non‐small cell lung cancer

## Abstract

**Background:**

Epidermal growth factor receptor (EGFR) activating mutations are usually associated with DNA damage repair (DDR) deficiency. However, the precise mechanism has remained elusive. In this study, we aimed to investigate whether EGFR exon 19 deletion mutation downstream signals contributed to DDR deficiency by downregulation of excision repair cross‐complementation group‐1 (ERCC1), a key factor in DDR, expression and function.

**Methods:**

We first measured cell survival, DNA damage (γ‐H2AX foci formation) and damage repair (ERCC1 and RAD51 foci formation) ability in response to DNA cross‐linking drug in EGFR exon 19 deletion and EGFR wild‐type cells separately. We then investigated the involvement of EGFR downstream signals in regulating ERCC1 expression and function in EGFR exon 19 deletion cells as compared with EGFR wild‐type ones.

**Results:**

We observed increased γ‐H2AX, but impaired ERCC1 and RAD51 nuclear foci formation in EGFR exon 19 deletion cells as compared with EGFR wild‐type ones treated with DNA cross‐linker. In addition, we identified that inhibition of EGFR exon 19 deletion signals increased ERCC1 expression, whereas blocked wild‐type EGFR signals decreased ERCC1 expression, on both mRNA and protein levels. Furthermore, EGFR exon 19 deletion downstream signals not only inhibited ERCC1 expression but also influenced ERCC1 foci formation in response to DNA cross‐linker.

**Conclusion:**

Our findings indicated that the aberrant EGFR exon 19 deletion signals were not only associated with decreased expression of ERCC1 but were also involved in impaired ERCC1 recruitment in response to DNA cross‐link damage, thereby providing us with more evidence for exploring the mechanism of DDR deficiency in EGFR mutant NSCLC.

## Introduction

Lung cancer is the leading cause of cancer‐related mortality worldwide and approximately 85% of lung cancers are non‐small cell lung cancer (NSCLC).^1^ Epidermal growth factor receptors (EGFR) are highly expressed in many malignant neoplasms. A total of 40%–60% NSCLC tumors show EGFR overexpression.^2^, ^3^ EGFR overexpression is usually associated with tumor invasion, metastasis and increased proliferation. EGFR has also been reported to promote DNA damage repair (DDR).^4^ Interestingly, NSCLC harboring EGFR activating mutations have previously been reported to be related to DDR deficiency which usually demonstrated as sensitivity to chemotherapy and radiotherapy, but the mechanism has remained elusive.^5^, ^6^

Recently, a link between EGFR and excision repair cross‐complementation group‐1 (ERCC1) has been reported. ERCC1 is a crucial factor involved in a number of DNA repair pathways in mammalian cells.^7^ It is essential for nucleotide excision repair (NER) and also has important roles in interstrand cross‐link (ICL) and double‐strand break (DSB) repair.^8^ Preliminary explorations have been made to investigate the relationship between EGFR and ERCC1. First, Liccardi *et al*. reported that wild‐type EGFR could translocate to the nucleus and bind with ERCC1 following DNA DSB induced directly by irradiation. This EGFR‐ERCC1 interaction was observed to be involved in DDR.^9^ Second, even without any DNA damage, Andrieux *et al*. determined that stimulation of wild‐type EGFR by its natural ligand epidermal growth factor (EGF) could increase ERCC1 expression through ERK pathway in hepatocyte and hepatocellular carcinoma cell lines.^10^

It is worth noting that NSCLC harboring EGFR activating mutations have been reported to demonstrate decreased levels of ERCC1 expression.^11^, ^12^ Most of these activating mutations occurred in EGFR gene exons 19 to 21 which encode the tyrosine kinase domain. Studies have revealed that mutations of kinase domains may facilitate EGFR dimerization, which in turn could promote kinase activity which gives rise to constitutive aberrant survival signals.^13^ Tyrosine kinase inhibitor (TKI) could inhibit this kinase activity effectively, block its downstream survival signals, and lead to cancer cell death.^14^ Thus, these survival signals raised by mutant EGFR have been reported to be oncogenic and tumor driver; however, the exact mechanism is still obscure. Considering that wild‐type EGFR downstream pathways could upregulate ERCC1 expression, we question whether the aberrant signals raised by mutant EGFR contribute to downregulation of ERCC1 in mutant EGFR NSCLC cells. If this is the case, we suggest that the decreased ERCC1 expression and impaired ERCC1 function induced by mutant EGFR signals may relate to tumorigenesis, as well as chemosensitivity, in NSCLC with EGFR activating mutations.

Evidence has revealed that the clinical outcomes of different EGFR mutation genotypes were varied. Exon 19 deletion is one of the most common activating EGFR mutations in NSCLC. In this study, we sought to explore the regulation of EGFR exon 19 deletion constitutive activating signals on ERCC1 expression and function in the process of DDR.

## Methods

### Cell lines

The human bronchial epithelial cell lines, BEAS‐2B and SAEC T1, were provided by Hanna K Lindberg (Finnish Institute of Occupational Health, Finland). NSCLC harboring EGFR exon 19 deletion cell line PC9 was purchased from Sigma‐Aldrich, Germany (90071810); and EGFR exon21 L855R mutation cell line HCC827 from the ATCC, USA (CRL‐2868). EGFR wild‐type NSCLC A549 and NCI‐H2126 cell lines were provided by Dr Emmy Verschuren (Institute for Molecular Medicine Finland, Finland). EGFR‐null NIH‐3T3 mouse embryonic fibroblasts (MEFs) were provided by the Oral cancer research group (University of Helsinki, Finland).

Culture conditions: PC9 cells were maintained in RPMI‐1640; A549, HCC827, and NIH‐3T3 cells were grown in Dulbecco's Modified Eagle Medium (DMEM); and NCI‐H2126 in DMEM/F12. Culture media supplements were 10% bovine growth serum (FBS) (HyClone SH30071.03), 10 mM HEPES (Gibco 15630–056), 2 mM L‐glutamine (Gibco 35050–038) and 1 mM sodium pyruvate (Gibco 11360–039). BEAS‐2B cells were cultured in LHC‐9 medium (Thermo Fisher 12680013). All the above cells were grown at 37°C in a humidified atmosphere with 5% CO_2_, and were in the logarithmic growth phase at the initiation of the experiments.

### Generation of MEFs expressing wild‐type and exon 19 deletion human EGFR

NIH‐3T3 cells were seeded in a six well plate at 10%–25% confluency. They were then transfected with the retroviral pBABE‐puromycin expression vector encoding human wild‐type EGFR or and mutant EGFR (exon 19 delL747‐E749 or exon21 L858R mutation) by retrovirus. Polybrene was added to each well at 8 μg/mL final concentration. The pBABE‐wild type EGFR plasmid, pBABE‐EGFR exon19 deletion plasmid, pBABE‐EGFR exon21 L858R plasmid and pBABE‐puro plasmid were obtained from the Addgene plasmid repository^15^, ^16^ (11011,11015,11012,1764). To select for stably transfected clones, puromycin‐containing media (final concentration 1 μg/mL) was added to the cells approximately 18–20 hours after infection.

### Treatments

Exponentially growing lung cancer cells, human bronchial epithelial cells and transfected NIH‐3T3 cells were seeded in six‐well plates, allowed to grow for 24 hours, and then collected for western blot analysis to measure protein expression. For inhibition of EGFR signals, cells were first cultured in serum‐supplemented media for 24 hours which was then replaced by media containing 0.1% FBS with or without 1 μM gefitinib, an EGFR‐TKI, (Iressa 250mg AstraZeneca) for further incubation.

To analyze ERCC1 dynamics in response to cisplatin, we placed exponentially growing NSCLC cells in a 96 well plate and treated the cells with 5 μM cisplatin (Sigma P4394) for 6, 12, 24, 48 hours and fixed for further analysis of the DNA damage and repair. In order to analyze the role of EGFR downstream pathways on ERCC1 recruitment in response to DNA damage, we first cultured lung cancer cells in low‐serum medium (0.1% FBS) containing 1 μM gefitinib for six hours, then treated the cells with 5 μM cisplatin in full medium for 6, 12, 24 and 48 hours.

### Western blot analysis

Cells were washed with ice‐cold phosphate‐buffered saline (PBS) and scraped immediately after adding 100 μL of cold RIPA buffer (Thermo 89 900) supplemented with protease inhibitor cocktail Set III, Animal Free (Calbiochem 535 140). Protein concentrations were measured using PieceTM BCA Protein Assay Kit (Thermo 23 227). For each sample, the same amount of total protein was added to a well of a 12% acrylamide gel and resolved by SDS‐PAGE. 1:150 dilution of antibody against human ERCC1 (Santa Cruz sc‐17 809), 1:1000 dilution of p44/42 MAPK (CST 4695), 1:2000 dilution of Phospho‐p44/42 MAPK (CST 4370) and 1:5000 of anti‐β‐actin (Sigma A5441) were used as primary antibodies. 1:5000 dilutions of HRP Donkey anti‐mouse or HRP Donkey anti‐rabbit IgG secondary antibodies (ThermoFisher A16017, A16035) were used. Proteins were detected with Clarity Western ECL Substrate (Bio‐Rad 170–5060) followed by Odyssey Fctoradiography (Li‐Cor 0720). The western blot images were analyzed using ImageJ software version 2.0.

### Immunofluorescence microscopy

Exponentially growing cells were fixed at different time points after treatment with 5 μM cisplatin (Sigma P4394) or 5 Gy gamma irradiation as follows. Cells were fixed with 2% PFA/PBS++ (PBS++: PBS with 1 mM CaCl2 and 0.5 mM MgCl2) for 10 minutes at 4°C, then permeabilized with 0.2% Triton X‐100 in PBS++ for 20 minutes. After blocking with staining buffer (0.5% BSA, 0.15%glycin, 0.1% Triton X‐100 in PBS++), they were exposed to primary antibodies against γ‐H2AX (Abcam ab22551; 1:1000 in staining buffer), ERCC1 (D‐10, Santa Cruz sc‐17 809; diluted 1:50 in 10% donkey serum in PBS) or RAD51 (Abcam ab22551, 1:500 in staining buffer) overnight at 4°C, subsequently incubated with secondary antibodies for one hour at room temperature (Alexa Fluor 488 donkey anti‐mouse, 1:500 in staining buffer, Invitrogen A‐21202 and Alexa Fluor 647 donkey anti‐rabbit, 1:500, Invitrogen A‐31573). Nuclear foci were scored using the Thermo Scientific CellInsight high content imaging system.

### Cell survival assays

Cell viability was measured by Trypan blue exclusion staining and tested by Countess™ automated cell counter (Invitrogen C10227).

### Statistical analysis

Statistical comparisons were calculated using Student's *t*‐test in SAS version 9. A *P*‐value of <0.05 was considered as statistically significant.

## Results

### Association of EGFR exon 19 deletion with DNA damage repair deficiency

**Figure 1:**
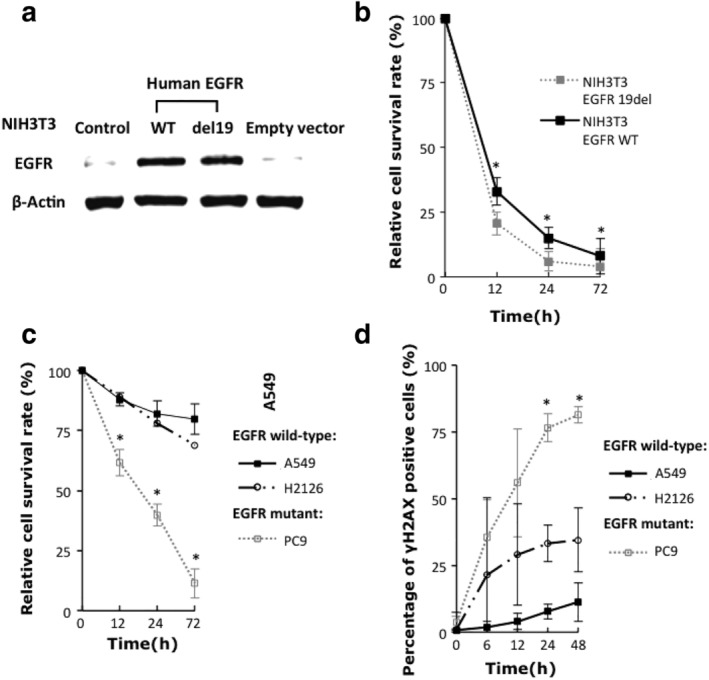
DNA damage repair (DDR) deficiency and DNA cross‐linker sensitivity of EGFR exon 19 deletion cells. (**a**) Western blot showed protein expression of human EGFR in NIH3T3 MEFs transfected with EGFR wild‐type or exon 19 deletion vectors. (**b**) Relative cell survival rate of wild‐type and del19 mutation EGFR transfected NIH3T3 MEFs cells after 5 μmol/L cisplatin treatment at different time points. (**c**) Relative cell survival rate of NSCLC cell lines after 5 μmol/L cisplatin treatment at different time points. (**d**) Percentage of γH2AX foci positive cells induced by 5 μmol/L cisplatin at different time points. All data represent mean ± SD based on two to three biology repeats. Student's *t*‐test was used for statistical analysis between EGFR exon 19 deletion and each EGFR wild‐type groups. * *P* < 0.05.

DDR deficiency is usually related to DNA damage drug sensitivity. In order to determine that DDR deficiency was associated with EGFR exon 19 deletion, we first studied cell survival in response to cisplatin (DNA cross‐linking drug) in both NSCLC cell lines and NIH3T3 MEFs expressing either wild‐type EGFR or EGFR harboring exon 19 deletion mutation. As shown in Figure 1a,b, NIH3T3 MEFs with exogenous EGFR exon 19 deletion were associated with lower cell‐survival fraction as compared with those of wild‐type EGFR. In lung cancer cell lines, we also observed that EGFR exon 19 deletion NSCLC cell line showed a lower survival rate than EGFR wild‐type ones after 5 μM cisplatin treatment (Fig 1c). In addition, γ‐H2AX, a marker for DNA double‐stranded breaks, was also observed. We found obviously increased γ‐H2AX foci formation in EGFR‐mutant PC9 cells as compared with wild‐type NSCLC cell lines in response to cisplatin (Fig 1d). These results together indicated that cells with EGFR exon 19 deletion were associated with deficiency of DNA damage repair.

### ERCC1 formation detected in EGFR exon 19 deletion cells

**Figure 2:**
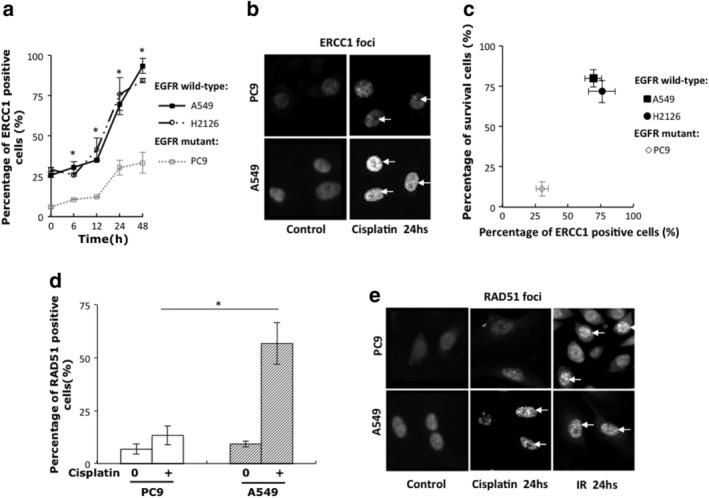
ERCC1 formation defects in EGFR exon 19 deletion cells in response to cross‐linking drug cisplatin in NSCLC cell lines. (**a**) Percentage of ERCC1 foci positive cells induced by 5μmol/L cisplatin at different time points. (**b**) Representative immunofluorescence microscopy images of nuclei with ERCC1 foci induced 24 hours after treatment with 5 μmol/L cisplatin. (**c**) Correlation of cisplatin survival rate with fraction of cells with induced ERCC1 foci. (**d**) Percentage of RAD51 foci positive cells induced 24 hours after 5 μmol/L cisplatin. (**e**) Representative immunofluorescence images of nuclei with Rad51 foci induced 24 hours after treatment with 5 μmol/L cisplatin or 5Gy gamma irradiation (IR). All data represent mean ± SD based on two to three biology repeats. Student's *t*‐test was used for statistical analysis between different groups. * *P* < 0.05.

ERCC1 is an essential protein in the NER pathway and Fanconi anemia pathway which is responsible for ICL unhooking and DNA DSB formation. To investigate the difference of ERCC1 protein function in EGFR wild‐type and exon 19 deletion NSCLC cells, we detected the formation of nuclear ERCC1 foci in response to DNA cross‐linking drug cisplatin by immunofluorescence. As shown in Figure 2a,b, we found a substantially increased number of nuclear ERCC1 foci 24 hours after cisplatin administration in wild‐type EGFR NSCLC cell lines, as compared with PC9 cells. In addition, our results showed that the ability of EGFR‐mutant and wild‐type NSCLC cell lines to form ERCC1 foci in response to cisplatin correlated with their survival after cisplatin treatment (Fig 2c).

Next, we determined whether the impaired Fanconi anemia pathway disrupted by ERCC1 affected the downstream homologous recombination (HR) repair pathway in EGFR exon 19 deletion NSCLC cells. RAD51, an important factor in HR pathway, was detected as a marker here. We observed that EGFR mutant PC9 cells exhibited defective formation of RAD51 foci in response to cisplatin as compared with EGFR wild‐type NSCLC cell lines (Fig 2d,e). However, we found the number of RAD51 foci increased significantly in both A549 and PC9 cells when the DSB caused directly by gamma irradiation (with no ICL unhooking process) (Fig 2e). Thus, it was possible to conclude that EGFR exon 19 deletion was associated with ERCC1 formation defects, which led to impaired response to DNA cross‐linking damage and sensitivity to cisplatin treatment.

### Aberrant regulation of ERCC1 by EGFR exon 19 deletion signals

**Figure 3:**
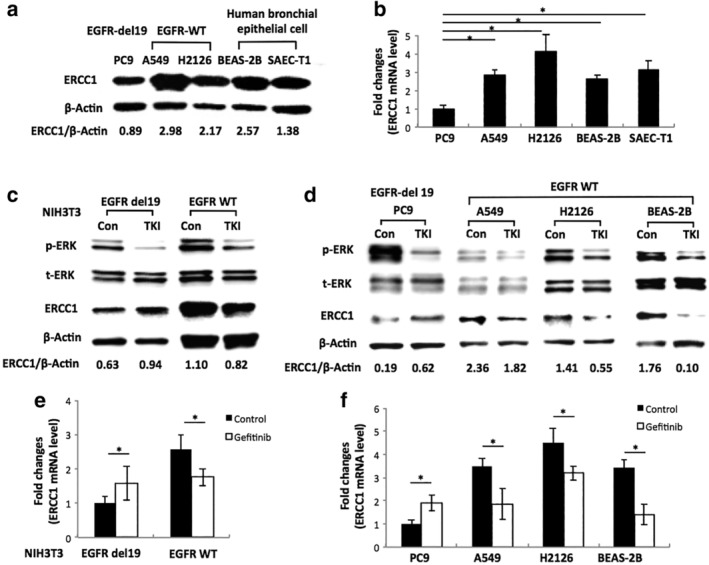
Regulation of ERCC1 by EGFR exon 19 deletion signals. (**a**) Western blot showing ERCC1 expression levels in NSCLC and human bronchial epithelial cell lines. (**b**) RT‐PCR showing mRNA level of ERCC1 expression in NSCLC and human bronchial epithelial cell lines. (**c**) Western blot analysis of ERCC1, phosphorylated ERK, total ERK protein expression in NIH3T3 MEFs transfected with different EGFR vectors, before and six hours after treated with gefitinib. (**d**) Western blot analysis of ERCC1, phosphorylated ERK, and total ERK protein expression in NSCLC cancer cell lines, before and six hours after treated with gefitinib. ERCC1 values were expressed as ratio to those of corresponding controls. (**e**) RT‐PCR analysis of ERCC1 mRNA expression of NIH3T3 MEFs transfected with wild‐type and 19 deletion EGFR vectors, before and six hours after treated with gefitinib. (**f**) RT‐PCR analysis of ERCC1 mRNA expression of NSCLC cell lines, before and six hours after treated with gefitinib. All data represent mean ± SD based on two to three biology repeats. Student's *t*‐test was used for statistical analysis between different groups. * *P* < 0.05.

In order to determine the relationship between EGFR exon 19 deletion and DNA damage repair gene ERCC1, we first screened ERCC1 expression in a panel of lung cell lines by western blot assay. We found both protein and mRNA expression level of ERCC1 in EGFR exon 19 deletion lung cancer cell line (PC9) were lower than EGFR wild‐type cancer cell lines and noncancerous human bronchial epithelial cell lines (Fig 3a,b). Then, we measured ERCC1 levels in NIH3T3 cells that stably expressed human EGFR wild‐type and exon 19 deletion vector. In these cell lines, we found that the protein expression ratio of ERCC1/β‐actin was lower in NIH3T3 cells harboring EGFR exon 19 deletion than cells with wild‐type EGFR (Fig 3c). These results demonstrated that EGFR exon 19 deletion was linked with lower expression of ERCC1.

Based on the above results, we further investigated our prediction that EGFR exon 19 deletion signals might take part in downregulation of ERCC1. To test this possibility, we first used EGFR‐TKI (gefitinib) to inhibit both mutant and wild‐type EGFR downstream signals, and then analyzed ERCC1 protein and mRNA expression levels in both NSCLC cell lines and in NIH3T3 MEFs. Total ERK and phosphorylated ERK protein were stained in the meantime to identify the inhibition effect of EGFR downstream signals. After EGFR inhibition, we found that NIH3T3 MEFs transfected with EGFR exon 19 deletion constructs demonstrated a higher expression of ERCC1 as compared with their corresponding controls; whereas NIH3T3 with wild‐type EGFR showed lower ERCC1 expression after gefitinib treatment (Fig 3c,e). In lung cancer cell lines, PC9 cells showed increased ERCC1 expression after gefitinib treatment. However, A549, H2126 and BEAS‐2B with wild‐type EGFR all showed decreased ERCC1 expression levels after EGFR signal inhibition (Fig 3d,f). Different from wild‐type EGFR regulation on ERCC1 expression, these results indicated EGFR exon 19 deletion downstream signals took part in downregulation of ERCC1 expression on both mRNA and protein levels.

### EGFR exon 19 deletion signals involved in ERCC1 blocking in response to cross‐linking drug

**Figure 4:**
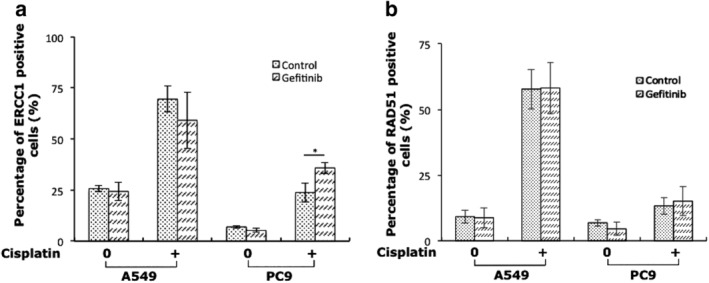
EGFR exon 19 deletion signals were involved in ERCC1 blocking in response to cross‐linking drug. (**a**) Average percentage of ERCC1 foci positive cells in gefitinib‐treated NSCLC cells, 24 hours after administration of 5 μmol/L cisplatin. (**b**) Average percentage of RAD51 foci positive cells in gefitinib‐treated NSCLC cells, 24 hours after administration of 5 μmol/L cisplatin. Cells were exposed to gefitinib six hours before adding cisplatin. All data represent mean ± SD based on two to three biology repeats. Student's *t*‐test was used for statistical analysis between different groups. * *P* < 0.05.

To determine whether the signal transduction pathways downstream of mutant EGFR were involved in impaired ERCC1 recruitment in response to cisplatin, we first treated EGFR exon 19 deletion PC9 cells, and together with EGFR wild‐type A549 as control, with gefitinib for six hours; then added cisplatin and observed the ERCC1 response. We found an increased percentage of ERCC1 foci after EGFR downstream signals blocked by gefitinib in response to cisplatin in PC9 cells; while there was decreased ERCC1 foci formation when EGFR signals were inhibited in A549 cells (Fig 4a). That is to say, EGFR exon 19 deletion downstream signals not only inhibited ERCC1 expression but also influenced ERCC1 foci formation which reflected DNA damage repair capacity.

In addition, we investigated whether exon 19 deletion EGFR signals impaired ERCC1 function, thereby blocking the downstream RAD51 foci rescue in response to cisplatin. However, we did not observe any stimulation of cisplatin‐induced RAD51 foci formation after inhibition of mutant EGFR pathway by gefitinib, nor did we find any suppression of RAD51 foci formation in EGFR wild‐type A549 cells upon inhibition of EGFR signals (Fig 4b). These results indicated that EGFR affects ERCC1 expression and function in a kinase dependent way; however, EGFR signals were not involved in the regulation of RAD51 recruitment in response to DNA damage. Thus, it was possible to conclude that EGFR exon 19 deletion signals might impair DDR on ERCC1 level, but not by disrupting RAD51 function, and other underlying mechanisms still need to be investigated.

## Discussion

NSCLC patients harboring EGFR activating mutations have been reported to be related to an increased response to platinum‐based chemotherapies as compared with EGFR wild‐type patients.^17^ Some studies have indicated that EGFR activating mutations may correlate with DNA damage repair deficiency.^18^ Different from wild‐type EGFR regulation on ERCC1 expression, in our study, we found EGFR exon 19 deletion signals could aberrantly downregulate ERCC1 expression. This result provided us with evidence to explain and further explore the mechanism of DDR deficiency in EGFR mutant NSCLC. In addition, EGFR exon19 deletion and exon21 L858R mutations are two common EGFR activating mutations. Interestingly, even though we found the regulation of EGFR exon 19 deletion on ERCC1, we did not find any regulatory relationship between EGFR L858R mutation signals and ERCC1 expression as demonstrated in our Supplementary data (Fig S1a,b). More and more clinical data revealed EGFR 19 deletion and 21 L858R mutation demonstrated different responses to treatments and clinical outcomes. It has been shown that patients harboring EGFR exon 19 deletion mutation respond better not only to EGFR‐TKIs but also to platinum‐based chemotherapy than patients with EGFR L858R mutation, but the underlying mechanism remains elusive.^19^, ^20^ With regard to our results, we suspected that downregulation of ERCC1 by EGFR exon 19 deletion signals impaired DNA damage repair ability could be the reason to explain that NSCLC with EGFR exon 19 deletion showed better response to platinum as compared with EGFR L858R mutation.

In our study, we found EGFR exon 19 deletion downstream signals not only inhibited ERCC1 expression but also influenced ERCC1 function in response to DNA damage. In mammalian cells, ERCC1 protein acts as an endonuclease in DNA damage repair process. It usually forms a highly conserved unhooking endonuclease complex with XPF that stabilizes both proteins for their roles in ICL repair and HR.^21^ In addition to ERCC1, our study also observed that XPF mRNA expression was decreased in EGFR exon 19 deletion NSCLC cells as compared with EGFR wild‐type ones (Fig S1c). Thus, we wondered whether the mutant EGFR signals influenced one unhooking endonuclease and other endonucleases were blocked as a result, or the mutant EGFR signals affected several ICL unhooking endonucleases together. A previous study revealed another endonuclease, FAN1, recruitment defect in mutant EGFR NSCLC cells in response to DNA ICL.^22^ FAN1 was a structure‐selective nuclease recruited to sites of cross‐link damage through binding the ubiquitinated FANCI‐FANCD2 complex.^23^ Notably, ERCC1 action on unhooking DNA ICL also depended on ubiquitination of FANCD2.^24^ However, it was reported that, in response to DNA cross‐linker, ubiquitinated FANCD2 level and FANCD2 foci formation were demonstrated to be similar in EGFR mutant cells as compared with EGFR wild‐type cells.^22^ With these data together, we indicated EGFR mutation affected DDR at the level of decreased DNA endonuclease expression and impaired endonuclease recruitment, which further reduced ICL incision and unhooking process.

Disrupted ICL unhooking pathway could affect aspects of downstream HR process in mutant EGFR NSCLC cells, which was detected in our study by impaired RAD51 foci formation in response to cisplatin. However, in our results, RAD51 foci accumulation did not decrease when DNA DSB was induced directly by gamma irradiation in EGFR mutant PC9 cells. These results, on the other hand, suggested that the DDR deficiency induced by EGFR exon 19 deletion was interrupted on the level of ICL unhooking and DNA incision. In addition, even though we found EGFR exon 19 deletion signals influenced ERCC1 foci formation in response to cisplatin, no stimulation of cisplatin‐induced RAD51 foci formation has been found after inhibition of the mutant EGFR pathway. A previous study revealed that transfection wild‐type EGFR into PC9 cells could rescue RAD51 foci formation, while overexpression of mutant EGFR into A549 wild‐type cells reduced RAD51 foci recruitment in response to cisplatin.^22^ Thus, it is possible to deduce that mutant EGFR not only disrupts ERCC1 recruitment but also some other damage repair factors in a kinase independent matter, so that one‐ended DSB substrate at the stalled fork cannot be produced properly which influence recruitment of RAD51 foci.

Even though we detected some regulation mechanism of EGFR exon 19 deletion signals on ERCC1 function, the exact entire regulation mechanism of EGFR exon 19 deletion on DDR deficiency is presently still far beyond our knowledge. In response to gamma irradiation, it was possible for wild‐type EGFR to translocate to the nucleus and then interact with ERCC1 to modulate DNA DSB^9^; while mutant EGFR, including exon 19 deletion and exon 21 L858R mutation, was unable to do so.^6^ In response to cisplatin, wild‐type EGFR could also translocate to the nucleus and bind to DNA protein kinase complex (DNA‐PK), which is an important protein in the NHEJ pathway. However, cells expressing EGFR L858R mutation could not translocate to the nucleus.^25^ Our study revealed a kinase dependent regulation of EGFR exon19 deletion signals on ERCC1 expression and function in response to cisplatin. Further in our Supplementary data, we also found EGFR with exon 19 deletion was also blocked in cytoplasm after cisplatin treatment (Fig S1d). Therefore, we supposed that the impaired EGFR nuclear localization was another reason which contributed to ICL repair deficiency in NSCLC harboring EGFR exon 19 deletion.

Furthermore, since ERCC1 endonuclease acted not only in ICL repair but also played a role in completion of HR following ICL repair,^26^ we considered that NSCLC cells with EGFR exon 19 deletion might be HR deficient and sensitive to PARP inhibitor. Preclinical research observed EGFR‐mutant cell lines exhibited olaparib (PARP inhibitor) sensitivity to a varying degree,^22^, ^27^ thus identification of NSCLC patients who could benefit from PARP inhibitor treatment is an important issue. According to our results, we supposed that EGFR exon 19 deletion might be a potential biomarker for PARP inhibitor. However, this hypothesis needs to be tested in NSCLC cell lines and clinical trials in the future.

## Disclosure

All authors declared no conflicts of interest of this manuscript.

## Supporting information


**Figure S1** (**a**) Western blot analysis of ERCC1, phosphorylated ERK, total ERK protein expression in HCC827 cells, before and six hours after treatment with gefitinib. (**b**)Western blot analysis of ERCC1, phosphorylated ERK, and total ERK protein expression in NIH3T3 MEFs transfected EGFR L858R mutation vectors, before and six hours after treatment with gefitinib. ERCC1 values were expressed as ratio to those of corresponding controls. (**c**)RT‐PCR showing mRNA level of XPF expression in NSCLC and human bronchial epithelial cell lines. (**d**)EGFR cellular localization following 18 hours treatment of cisplatin in EGFR wild‐type and EGFR mutant NSCLC cell lines. All data represent mean ± SD based on two to three biology repeats. Student's *t*‐test was used for statistical analysis between different groups. * *P* < 0.05.

## References

[1] Siegel RL , Miller KD , Jemal A . Cancer statistics, 2016. CA Cancer J Clin 2016; 66 (1): 7–30. 26742998 10.3322/caac.21332

[2] Rivera F , Vega‐Villegas ME , Lopez‐Brea MF et al. Current situation of panitumumab, matuzumab, nimotuzumab and zalutumumab. Acta Oncol 2008; 47 (1): 9–19. 18097777 10.1080/02841860701704724

[3] Crombet T , Torres L , Neninger E et al. Pharmacological evaluation of humanized anti‐epidermal growth factor receptor, monoclonal antibody h‐R3, in patients with advanced epithelial‐derived cancer. J Immunother 2003; 26 (2): 139–48. 12616105 10.1097/00002371-200303000-00006

[4] Bai J , Guo XG , Bai XP . Epidermal growth factor receptor‐related DNA repair and radiation‐resistance regulatory mechanism: A mini‐review. Asian Pac J Cancer Prev 2012; 13 (10): 4879–81. 23244074 10.7314/apjcp.2012.13.10.4879

[5] Das AK , Sato M , Story MD et al. Non‐small‐cell lung cancers with kinase domain mutations in the epidermal growth factor receptor are sensitive to ionizing radiation. Cancer Res 2006; 66 (19): 9601–8. 17018617 10.1158/0008-5472.CAN-06-2627

[6] Das AK , Chen BP , Story MD et al. Somatic mutations in the tyrosine kinase domain of epidermal growth factor receptor (EGFR) abrogate EGFR‐mediated radioprotection in non‐small cell lung carcinoma. Cancer Res 2007; 67 (11): 5267–74. 17545606 10.1158/0008-5472.CAN-07-0242

[7] Gossage L , Madhusudan S . Current status of excision repair cross complementing‐group 1 (ERCC1) in cancer. Cancer Treat Rev 2007; 33 (6): 565–77. 17707593 10.1016/j.ctrv.2007.07.001

[8] McNeil EM , Melton DW . DNA repair endonuclease ERCC1‐XPF as a novel therapeutic target to overcome chemoresistance in cancer therapy. Nucleic Acids Res 2012; 40 (20): 9990–10004. 22941649 10.1093/nar/gks818 PMC3488251

[9] Liccardi G , Hartley JA , Hochhauser D . Importance of EGFR/ERCC1 interaction following radiation‐induced DNA damage. Clin Cancer Res 2014; 20 (13): 3496–506. 24780295 10.1158/1078-0432.CCR-13-2695

[10] Andrieux LO , Fautrel A , Bessard A et al. GATA‐1 is essential in EGF‐mediated induction of nucleotide excision repair activity and ERCC1 expression through ERK2 in human hepatoma cells. Cancer Res 2007; 67 (5): 2114–23. 17332341 10.1158/0008-5472.CAN-06-3821

[11] Gandara DR , Grimminger P , Mack PC et al. Association of epidermal growth factor receptor activating mutations with low ERCC1 gene expression in non‐small cell lung cancer. J Thorac Oncol 2010; 5 (12): 1933–8. 20975603 10.1097/JTO.0b013e3181fd418d

[12] Ren S , Chen X , Kuang P et al. Association of EGFR mutation or ALK rearrangement with expression of DNA repair and synthesis genes in never‐smoker women with pulmonary adenocarcinoma. Cancer 2012; 118 (22): 5588–94. 22569898 10.1002/cncr.27603

[13] Shan Y , Eastwood MP , Zhang X et al. Oncogenic mutations counteract intrinsic disorder in the EGFR kinase and promote receptor dimerization. Cell 2012; 149 (4): 860–70. 22579287 10.1016/j.cell.2012.02.063

[14] Ji H , Li D , Chen L et al. The impact of human EGFR kinase domain mutations on lung tumorigenesis and in vivo sensitivity to EGFR‐targeted therapies. Cancer Cell 2006; 9 (6): 485–95. 16730237 10.1016/j.ccr.2006.04.022

[15] Greulich H , Chen TH , Feng W et al. Oncogenic transformation by inhibitor‐sensitive and ‐resistant EGFR mutants. PLOS Med 2005; 2 (11): e313. 10.1371/journal.pmed.0020313 PMC1240052 16187797

[16] Morgenstern JP , Land H . Advanced mammalian gene transfer: High titre retroviral vectors with multiple drug selection markers and a complementary helper‐free packaging cell line. Nucleic Acids Res 1990; 18 (12): 3587–96. 2194165 10.1093/nar/18.12.3587 PMC331014

[17] Mok TS , Wu YL , Thongprasert S et al. Gefitinib or carboplatin‐paclitaxel in pulmonary adenocarcinoma. N Engl J Med 2009; 361: 947–57. 19692680 10.1056/NEJMoa0810699

[18] Birkelbach M , Ferraiolo N , Gheorghiu L et al. Detection of impaired homologous recombination repair in NSCLC cells and tissues. J Thorac Oncol 2013; 8: 279–86. 23399959 10.1097/JTO.0b013e31827ecf83 PMC3573529

[19] Yamashita F , Azuma K , Yoshida T et al. Prognostic value of EGFR mutation and ERCC1 in patients with non‐small cell lung cancer undergoing platinum‐based chemotherapy. PLOS One 2013; 8 (8): e71356. 23940741 10.1371/journal.pone.0071356 PMC3734014

[20] Zhang Y , Sheng J , Kang S et al. Patients with exon 19 deletion were associated with longer progression‐free survival compared to those with L858R mutation after first‐line EGFR‐TKIs for advanced non‐small cell lung cancer: A meta‐analysis. PLOS One 2014; 9 (9): e107161. 25222496 10.1371/journal.pone.0107161 PMC4164616

[21] Volker M , Moné MJ , Karmakar P et al. Sequential assembly of the nucleotide excision repair factors in vivo. Mol Cell 2001; 8 (1): 213–24. 11511374 10.1016/s1097-2765(01)00281-7

[22] Pfäffle HN , Wang M , Gheorghiu L et al. EGFR‐activating mutations correlate with a Fanconi anemia‐like cellular phenotype that includes PARP inhibitor sensitivity. Cancer Res 2013; 73 (20): 6254–63. 23966292 10.1158/0008-5472.CAN-13-0044 PMC3823187

[23] Liu T , Ghosal G , Yuan JS et al. FAN1 acts with FANCI–FANCD2 to promote DNA interstrand cross‐link repair. Science 2010; 329 (5992): 693–6. 20671156 10.1126/science.1192656

[24] Klein Douwel D , Boonen RA , Long DT et al. XPF‐ERCC1 acts in unhooking DNA interstrand crosslinks in cooperation with FANCD2 and FANCP/SLX4. Mol Cell 2014; 54 (3): 460–71. 24726325 10.1016/j.molcel.2014.03.015 PMC5067070

[25] Liccardi G , Hartley JA , Hochhauser D . EGFR nuclear translocation modulates DNA repair following cisplatin and ionizing radiation treatment. Cancer Res 2011; 71 (3): 1103–14. 21266349 10.1158/0008-5472.CAN-10-2384 PMC3033323

[26] Al‐Minawi AZ , Lee YF , Håkansson D et al. The ERCC1/XPF endonuclease is required for completion of homologous recombination at DNA replication forks stalled by inter‐strand cross‐links. Nucleic Acids Res 2009; 37 (19): 6400–13. 19713438 10.1093/nar/gkp705 PMC2770670

[27] Li N , Feng L , Liu H et al. PARP inhibition suppresses growth of EGFR‐mutant cancers by targeting nuclear PKM2. Cell Rep 2016; 15 (4): 843–56. 27149849 10.1016/j.celrep.2016.03.070 PMC5063668

