# Supplementary figures and images for: EGFR exon 19‐deletion aberrantly regulate ERCC1 expression that may partly impaired DNA damage repair ability in non‐small cell lung cancer
Source: Thorac Cancer. 2019 Dec 25;11(2):277–85. doi: 10.1111/1759-7714.13253 (PMC6996978; doi:10.1111/1759-7714.13253)

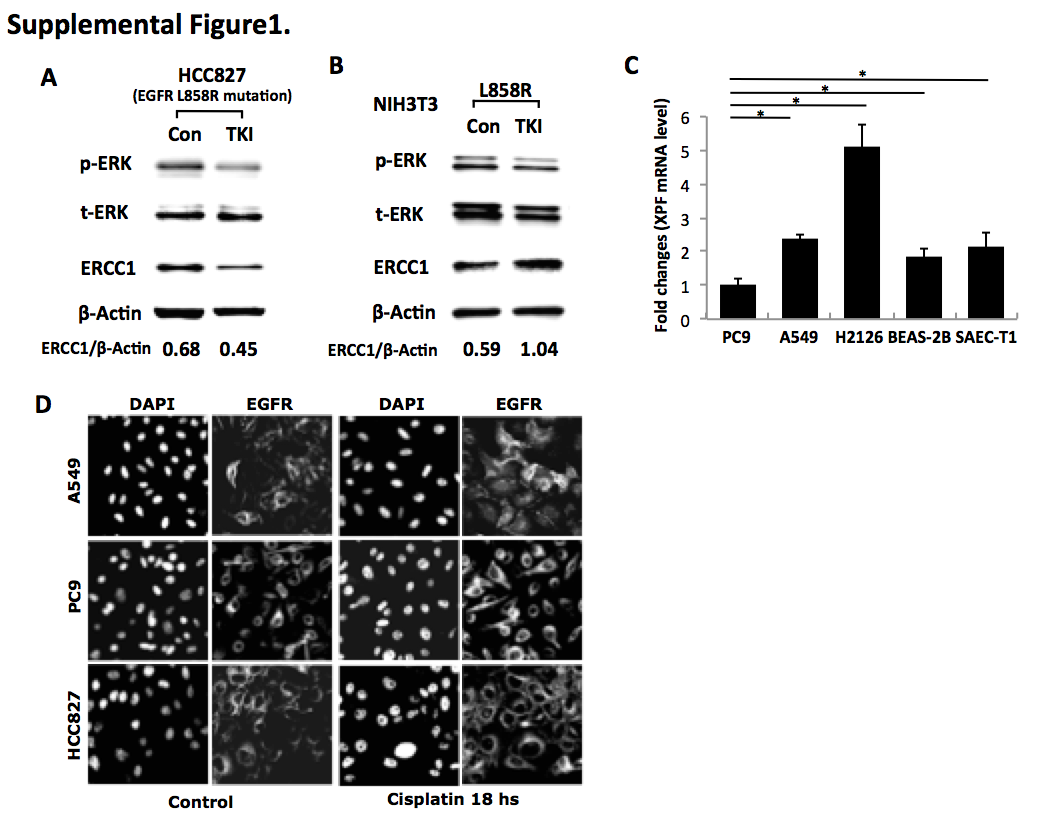

Supplement: Supplementary file 1 — Figure S1 (a) Western blot analysis of ERCC1, phosphorylated ERK, total ERK protein expression in HCC827 cells, before and six hours after treatment with gefitinib. (b)Western blot analysis of ERCC1, phosphorylated ERK, and total ERK protein expression in NIH3T3 MEFs transfected EGFR L858R mutation vectors, before and six hours after treatment with gefitinib. ERCC1 values were expressed as ratio to those of corresponding controls. (c)RT‐PCR showing mRNA level of XPF expression in NSCLC and human bronchial epithelial cell lines. (d)EGFR cellular localization following 18 hours treatment of cisplatin in EGFR wild‐type and EGFR mutant NSCLC cell lines. All data represent mean ± SD based on two to three biology repeats. Student's t‐test was used for statistical analysis between different groups. * P < 0.05. [file TCA-11-277-s001.png]
